# Nerve transfer for restoration of lower motor neuron-lesioned bladder function. Part 1: attenuation of purinergic bladder smooth muscle contractions

**DOI:** 10.1152/ajpregu.00299.2020

**Published:** 2021-03-24

**Authors:** Nagat Frara, Dania Giaddui, Alan S. Braverman, Danielle S. Porreca, Justin M. Brown, Michael Mazzei, Ida J. Wagner, Michel A. Pontari, Ekta Tiwari, Courtney L. Testa, Daohai Yu, Lucas J. Hobson, Mary F. Barbe, Michael R. Ruggieri

**Affiliations:** ^1^Department of Anatomy and Cell Biology, Lewis Katz School of Medicine, Temple University, Philadelphia, Pennsylvania; ^2^Department of Neurosurgery, Massachusetts General Hospital, Boston, Massachusetts; ^3^Department of Surgery, Lewis Katz School of Medicine, Temple University Hospital, Philadelphia, Pennsylvania; ^4^Department of Urology, Lewis Katz School of Medicine, Temple University Hospital, Philadelphia, Pennsylvania; ^5^Department of Clinical Sciences, Lewis Katz School of Medicine, Temple University, Philadelphia, Pennsylvania; ^6^Department of Electrical and Computer Engineering, College of Engineering, Temple University, Philadelphia, Pennsylvania

**Keywords:** atropine-sensitive, cholinergic, ex vivo bladder smooth muscle strip contractility, nerve-evoked contractions, purinergic

## Abstract

This study determined the effect of pelvic organ decentralization and reinnervation 1 yr later on the contribution of muscarinic and purinergic receptors to ex vivo, nerve-evoked, bladder smooth muscle contractions. Nineteen canines underwent decentralization by bilateral transection of all coccygeal and sacral (S) spinal roots, dorsal roots of lumbar (L)7, and hypogastric nerves. After exclusions, 8 were reinnervated 12 mo postdecentralization with obturator-to-pelvic and sciatic-to-pudendal nerve transfers then euthanized 8-12 mo later. Four served as long-term decentralized only animals. Controls included six sham-operated and three unoperated animals. Detrusor muscle was assessed for contractile responses to potassium chloride (KCl) and electric field stimulation (EFS) before and after purinergic receptor desensitization with α, β-methylene adenosine triphosphate (α,β-mATP), muscarinic receptor antagonism with atropine, or sodium channel blockade with tetrodotoxin. Atropine inhibition of EFS-induced contractions increased in decentralized and reinnervated animals compared with controls. Maximal contractile responses to α,β-mATP did not differ between groups. In strips from decentralized and reinnervated animals, the contractile response to EFS was enhanced at lower frequencies compared with normal controls. The observation of increased blockade of nerve-evoked contractions by muscarinic antagonist with no change in responsiveness to purinergic agonist suggests either decreased ATP release or increased ecto-ATPase activity in detrusor muscle as a consequence of the long-term decentralization. The reduction in the frequency required to produce maximum contraction following decentralization may be due to enhanced nerve sensitivity to EFS or a change in the effectiveness of the neurotransmission.

## INTRODUCTION

Pathological conditions that disrupt bladder innervation, such as lower motor neuron lesions (1), may alter muscarinic and purinergic components and receptors taking part in detrusor contractility (2–8). A systematic review of surveys administered to people with spinal cord injury highlighted that retrieval of bladder and bowel function is a top recovery priority (9). Selective pharmacological manipulations might be an appropriate management for lower motoneuron lesioned neurogenic bladder disorders, which would result in an improvement of the quality of life (1).

The urinary bladder receives mixed sensory and motor innervation through hypogastric nerves carrying sympathetic axons and pelvic nerves carrying parasympathetic axons. The pelvic plexus integrates these sympathetic and parasympathetic fibers with ganglia that originate within the plexus (10). In most species, including humans, intramural ganglia in the bladder wall receive projections from the pelvic plexus ganglia, as well as from hypogastric and pelvic nerves (11, 12). Post ganglionic, parasympathetic nerves are responsible for detrusor contraction by the release of acetylcholine (ACh), the main excitatory neurotransmitter acting on muscarinic receptors (13), and adenosine triphosphate (ATP), a coneurotransmitter acting on purinergic receptors (14). There is increasing evidence that the autonomic nervous system develops plasticity to compensate for some of the loss of innervation that occurs following trauma, surgery, or diseases through changes in the expression of neurotransmitters and cotransmitters released from the remaining nerves (15). Pharmacological studies reveal that the contribution of cholinergic and purinergic neurotransmission to the control of bladder function is variable among different species. Although the cholinergic and purinergic proportions of parasympathetic cotransmission are almost equal in rodents and ATP contributes to a prominent portion of nerve-evoked bladder contractions in canine bladder, it is mainly cholinergic in normal human bladder (16).

As part of a larger project to develop a strategy for surgical reinnervation following long-term lower motoneuron injury, this study investigates the effects of extensive sensory and motor decentralization and reinnervation on detrusor muscle contractility using ex vivo contractile responses as a measure of detrusor smooth muscle function. In addition, it determines if the surgical reinnervation of the bladder after 1 yr of lower spinal root decentralization changes the contribution of muscarinic or purinergic receptors mediating nerve-evoked contractions in the detrusor muscle. Establishment of the canine model and interim pilot study results have been published previously (17). Understanding the neuronal mechanisms that underlie changes in bladder innervation after spinal root injury and surgical reinnervation would provide insight into development of possible pharmacological treatment strategies that could promote recovery and maintenance of bladder function.

## METHODS

### Animals

Studies were approved by the Institutional Animal Care and Use Committee according to guidelines of the National Institute of Health for the Care and Use Laboratory Animals and the United States Department of Agriculture and the Association for Assessment and Accreditation of Laboratory Animal Care. Results are reported for 21 female mixed-breed hound dogs, 6–8 mo old, weighing 20–25 kg (Marshall BioResources, North Rose, NY). This manuscript is Part 1 of a series and there is some overlap with the pilot study of this series (17). Specifically, squat-and-void data from our pilot study is included in this report for three of the reinnervated, one of the decentralized, and six of the sham-operated animals.

### Original Design and Necessary Modifications of the Project Plan

**Figure 1.**
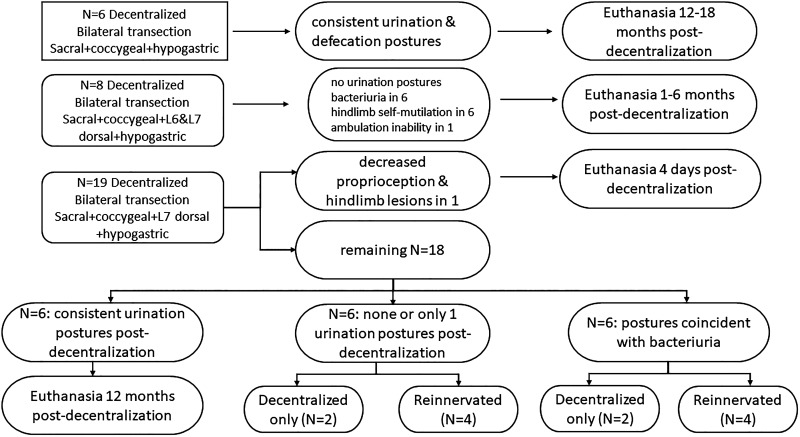
Flowchart for the decentralization and reinnervation surgeries.

Figure 1 is a flowchart of the decentralized and reinnervated animals. Based on a preliminary power analysis of data from our previous short-term decentralization and reinnervation studies (18, 19), the original project design was intended to include 12 animals in each of the four experimental surgical groups: *group 1*, decentralized and immediately reinnervated; *group 2*, 12 mo decentralized then reinnervated; *group 3*, 12 mo decentralized controls; and *group 4*, 6 mo sham decentralized controls. Due to unanticipated outcomes regarding the proportion of decentralized animals that showed no urination postures, the number of animals per group was necessarily reduced. Decentralization was initially performed by bilateral transection of all sacral and coccygeal spinal roots and bilateral hypogastric nerve transection. Monthly video surveillance of urination postures in the canines’ housing cages during post decentralization follow-up of the first three dogs in each of the two long-term decentralized groups confirmed that this surgery did not prevent urination or defecation postures. Because retrograde dye labeling from the bladder to the dorsal root ganglia (DRG) documented sensory innervation of the bladder from both lumbar (L)6 and L7 DRGs (17), as well as the well-known sacral DRGs, we modified the design by studying two groups of progressively more extensively decentralized animals: *group 1*, all roots caudal to L7, hypogastric nerves, and L7 dorsal roots, and *group 2*, all roots caudal to L7, hypogastric nerves, and both L6 and L7 dorsal roots. Although none of the eight animals with the additional the L6 and L7 dorsal root transections showed urination postures in their housing cages, they suffered from multiple postoperative complications, including bacteriuria in six, hind limb self-mutilation in six, and ambulation inability in one that mandated their euthanasia 1–6 mo after decentralization surgery, before our ability to perform nerve transfer surgery or retrograde neurotracing studies.

A total of 19 animals were decentralized by transection of all roots caudal to L7, hypogastric nerves, and L7 dorsal roots. One of the 19 developed decreased proprioception with hindlimb lesions requiring euthanasia 4 days after decentralization. Six of the remaining 18 were excluded because they showed consistent urination postures in their housing cages over 6 mo postoperatively during monthly, 24-h observations, indicating that they were not functionally decentralized, and consequently, were euthannized. Of the remaining 12 animals, six showed none or only one urination postures in their housing cages for 12 mo after decentralization. The other six showed these postures only coincident with culture confirmed bacteriuria that disappeared with clearing of the bacteriuria with appropriate antibiotic treatment. Those 12 animals were considered functionally decentralized and therefore, were included in this study.

It is worth noting that it is not possible to completely decentralize the bladder because some afferents that originate from pathways other than the lower lumbosacral and hypogastric nerves that were transected would still contribute to bladder sensation. With decentralization surgeries, our goal was to minimize the urination postures as much as possible and observe the return of these postures as a measurement of functional sensory reinnervation. As mentioned in the pilot study of this series (17), observation of the squat-and-void behaviors despite decentralization indicates that they either retained or regained some bladder sensation following decentralization, perhaps due to activation of silent C fibers by the bacteriuria, postoperative sensory nerve sprouting resulting in spontaneous bladder sensory reinnervation, or variations in bladder sensory innervation, as we previously reported (20). In a group of eight dogs, the additional transection of L6 dorsal roots eliminated urination postures in all eight dogs, indicating sensory innervation from lower lumbar dorsal roots. There was no intentional bias. The functionally decentralized dogs were not randomized. We chose animals that did not show the micturition behavior for reinnervation. Once an *n* of 8 reinnervated animals was reached, the remainder served as decentralized only animals. At 12 mo post decentralization, four out of six animals in each of these two functionally decentralized subcategories (those showing no or only one urination posture or those in which bacteriuria induced urination postures) were assigned to nerve transfer reinnervation surgery and two of each group of six were placed into the long-term decentralized group. Accordingly, the current study reports results from 21 animals: eight functionally decentralized for 9–13 mo then reinnervated (ObNT Reinn) and followed for 7–12 mo, four decentralized for 11–21 mo (Decentralized), and nine normal controls; six sham-operated and three unoperated (Sham/Unop).

### Decentralization and Nerve Transfer Surgeries

Animals were fasted the day before surgery and received 20 mg/kg iv cefazolin with redosing every 4 h until completion of the procedure. Postoperative antibiotic prophylaxis included 30 mg/kg cephalexin PO, twice a day for 5 days. Before any surgical procedure, dogs were sedated with propofol (6 mg/kg iv) to allow endotracheal intubation, and then anesthesia was maintained using isoflurane (2%–4% maximum alveolar concentration) with oxygen as the carrier gas. Catheterization was performed for placement of dual balloon catheters for monitoring of urethral and bladder pressures (21). All animals, but the unoperated controls, underwent a laminectomy of L6-sacral (S)2 vertebrae to expose the lower spinal cord and spinal roots. L7 and S1–3 roots were identified before transection. In all but the sham-operated and unoperated control animals, decentralization was performed by bilateral transection of all dorsal and ventral roots caudal to L7 and L7 dorsal roots. Then, to ensure complete separation, 10–15-mm sections from each transected root or nerve was removed. In eight of the decentralized animals, the spinal ganglia were completely removed, whereas, in the other four, the spinal ganglia remained intact with their connections to the spinal cord removed. One of the four with their ganglia intact was placed in the decentralized-only control group and the remaining three were reinnervated. Hypogastric nerves were accessed via abdominal surgery and were bilaterally transected, with 10–15-mm length excised, from their proximal emergence from the inferior mesenteric ganglion to their distal entrance into the pelvic plexus. All animals underwent tail amputation at the end of the procedure to prevent self-mutilation of the now decentralized tail.

Sham-operated controls underwent lumbosacral laminectomy, nerve root identification via electrical stimulation without root transection, and abdominal opening with identification of hypogastric nerves. Because no differences were observed between the sham-operated and unoperated controls in this or prior studies (20, 22), these animals were combined into one normal control group.

At 1 yr postdecentralization, eight animals underwent bilateral obturator nerve transfer to the anterior vesicle branches of their pelvic nerves, and then an additional 8–12 mo (average of 10 months) post reinnervation recovery. The remaining four served as long-term decentralized only animals. For the nerve transfer surgery, eight animals were anesthetized and catheterized with balloon catheters. Obturator nerves (a primarily somatic motor nerve whose natural path runs very close to the pelvic plexus) were accessed via abdominal surgery, identified, and divided longitudinally. Approximately 75% of the obturator nerve was left intact to retain innervation of hind limb adductor muscles. The other quarter of the fascicles were transected, transferred, and the proximal end was sutured end-to-end to the distal transected anterior vesical branch of the pelvic nerve, bilaterally. Axoguard nerve connectors (Axogen Corp, Alachua, FL) were used to maintain transferred nerve coaptation and to reinforce the coaptation site which was covered in Tisseel fibrin sealant (Baxter, Deerfield, IL).

For functional reinnervation of the external urethral and anal sphincters, a redundant branch of the sciatic nerve was transferred to branches of the pudendal nerve that induced urethral and anal sphincter contractions with intraoperative electrical stimulation. The effects of the pudendal nerve transfer on the urethral and anal sphincters are part of another study and are not presented in this manuscript. Portions have been previously reported (17).

### Postoperative Care

For each surgical procedure, buprenorphine (0.03–0.05 mg/kg) was administered subcutaneously, twice a day for 2 days post-op. The Credé maneuver was performed on all decentralized animals twice daily. Because of the S1–S3 root transection and loss of both pelvic and pudendal nerve function, the decentralized animals were incontinent of urine and thus a Credé maneuver was not generally required for bladder emptying (although it was performed). Regarding potential obturator nerve transection gait changes, sometimes during recovery, the reinnervated animals’ hind legs would splay outward laterally, but this always resolved within a week and sometimes even within 24 h of the nerve transfer surgery. The frequency of squat-and-void postures, defined by the position exhibited by female canines during urination, was recorded for 24 h at monthly intervals pre- and postoperatively. Sterile catheterized urine specimens, collected generally at monthly intervals, was assessed by a commercial veterinary clinical laboratory (Antech USA Laboratory Diagnostics) for routine urinalysis, bacterial species identification and, if culture positive, antibiotic sensitivity. Animals with positive urine cultures received antibiotic treatment based on the antibiotic sensitivity of the organisms cultured from the urine until repeated catheterized urine specimens were culture negative.

### Ex Vivo Bladder Smooth Muscle Strip Contractility Studies

Immediately before euthanasia, the bladder was removed and washed in Tyrode’s buffer composed of 125 mM NaCl, 27 mM KCl, 4.2 mM NaH_2_PO_4_, 1.8 mM CaCl_2_, 5 mM MgCl_2_, 23.8 mM NaHCO_3_, and 5.6 mM dextrose. A full-thickness specimen of the dorsal midbladder between the dome and 1 cm above the bladder neck was removed, immersed in Custodiol HTK organ transport media, composed of 15 mM NaCl, 9 mM KCl, 1 mM potassium hydrogen 2-ketoglutarate, 4 mM MgCl_2_, 18 mM histidine HCl, 180 mM histidine, 2 mM tryptophan, 30 mM mannitol, and 0.015 mM CaCl_2_, and stored on ice at 4°C for muscle strip contractility studies to be performed the following day. Mucosa-denuded bladder muscle strips were suspended in muscle baths containing 10 mL of Tyrode’s solution, and maintained aerated with 95% O_2_ and 5% CO_2_ at 37°C. Strips were initially stretched slowly to 2.0 g of isometric tension and allowed to relax to ∼1 g of basal tension. Contractile responses were monitored with isometric force transducers, as described previously (21). Strips were treated with isotonic Tyrode’s solution containing 120 mM potassium chloride (KCl) and the contractile responses induced by KCl were measured. Electric field stimulations (EFS) were delivered to each strip using a Grass S88 stimulator (Natus Neurology, Inc., Warwich, RI) interfaced with a Stimu-Splitter II (Med-Lab Instruments, Loveland, CO) power amplifier and LabChart software (ADInstruments). The neuronal basis of the responses was verified by 1 µM tetrodotoxin (TTX) blockade. Frequency-response curves were generated at 12 V, 1 ms pulse duration, and a series of increasing frequencies (2, 5, 12, 20, and 30 Hz) to evaluate nerve-evoked contractions. In separate strips, EFS-induced contractions were determined in the presence of the vehicle (H_2_O), 10 µM α,β-mATP, or 1 µM atropine; added at 1/100 the bath volume from 100× stock solutions to assess the contribution of cholinergic and purinergic receptors mediating nerve-evoked contractions, respectively. The contractile response was assessed by performing frequency-response curves before and after drug exposure. Responses are expressed as percentages of maximal EFS-induced contraction of the frequency response curve. Half maximal effective frequency (EF_50_) values were determined for each strip using a sigmoidal curve fit of the data (Origin; OriginLab Corp., Northampton, MA), which were used to compare the sensitivity of EFS-induced contractions in different groups.

The physical dimensions and weight of the muscle strips were comparable across groups. No differences in the strip length expressed as means ± 95% confidence intervals (CI): 10.86 (95% CI: 10.15–11.57) in male sham (*N* = 1, *n* = 46), 9.14 (95% CI: 8.55–11.27) in Decentralized (*N* = 3, *n* = 96) and 9.21 (95% CI: 8.40–10.04) in ObNT Reinn (*N* = 5, *n* = 160) bladders. Also, no differences in the strip weight expressed as means ± 95% CI: 12.90 (95% CI: 11.51–14.31) in male sham, 18.64 (95% CI: 17.0–20.26) in Decentralized and 16.40 (95% CI: 14.75–18.06) in ObNT Reinn bladders. Because the muscle strips were suspended along their length between two spring wire clips (catalog no. 158802, Radnoti LLC, Covina, CA), the portion of the smooth muscle that induces the force for all strips is nearly identical between different strips.

### Statistical Analyses

Statistical analyses were performed using SAS v. 9.4 (SAS Institute Inc., Cary, NC). Data are presented as means ± 95% CIs. The calculated *P* value is based on the number of animals (not on replicate data). These *P* values were adjusted more stringently in the post hoc multiple comparison tests, and these adjusted *P* values are reported. Because this study did not test a prespecified statistical null hypothesis, this makes it exploratory, therefore the calculated *P* values are interpreted as descriptive, not as hypothesis testing.

When comparing one variable across the three experimental surgical groups (e.g., responses to KCl, α,β-mATP, EFS, and EF_50_s), a mixed-effects linear regression model was used to take into account the correlation between data points from multiple strips per each animal used in each group, followed by Dunnett’s post hoc multiple comparisons to determine differences between the two experimental groups versus control. For the analyses of the data with two factors (i.e., surgical group and electrical stimulation frequency or surgical group and drug treatment) and unequal numbers per group, a mixed-effects linear regression model with an interaction term between the two factors as well as adjustment for the pretreatment measurement of the study end point, when appropriate, was fitted to the data. This was done to take into consideration the correlation between data points from multiple strips per animal as before and repeated measures across electrical stimulation frequencies or drug treatment groups. This was followed by Dunnett’s multiple-comparison post hoc tests to determine differences between the two experimental surgical groups versus control for each given frequency or between-drug treatment groups versus vehicle for each experimental surgical group.

## RESULTS

### Observations following Surgery

Animals were monitored for any squat-and-void postures after decentralization. We define the squat-and-void posture as the position exhibited by female dogs during urination. Some decentralized animals continuously leaked urine and some exhibited unusual postures that were judged to be intermediate between urination and defecation postures with longer duration as reported in the pilot study of this series (17), and those postures were coincident with culture-confirmed bacteriuria, which disappeared with antimicrobial treatment. Therefore, to emphasize our observations and to differentiate between the two types of postures, we use “squat-and-void urination behaviors” for the usual urination postures. As described in the pilot study, 5 of the 12 decentralized animals showed no squat-and-void urination behaviors during the monthly, 24-h, postoperative observation periods and one only showed one behavior on two occasions (17). The other six decentralized animals showed increased squat-and-void behaviors 1–8 mo after decentralization coincident with culture confirmed bacteriuria. These behaviors were no longer observed after their bacteriuria was cleared with appropriate antibiotic treatment. Although throughout the study, none of the animals in any of the treatment groups had a body temperature over 39.5°C indicative of a urinary tract infection (UTI) induced fever, all instances of bacteriuria were treated with antibiotics based on the culture sensitivity until repeat urine cultures became negative. No catheterized urine specimens from animals collected before any surgery were culture positive and no sham-operated animals had any culture positive catheterized urine specimens. Urine samples were collected during the awake bladder filling that was performed every 3 wk starting 1 mo postdecentralization and postreinnervation and continued over time until euthanasia. All of the Decentralized and ObNT Reinn animals had multiple instances of culture confirmed bacteriuria. Over the course of the postoperative period, Decentralized only animals had an average of 12 ± 8 (means ± SD) positive urine cultures episodes, 58% ± 40% of which were associated with urinalysis findings of occult blood or >4 white or red blood cells per high power field, consistent with an active UTI. ObNT Reinn animals had an average of 13 ± 3 culture positive urine cultures, 73% ± 21% of which were associated with urinalysis findings of occult blood or >4 white or red blood cells per high-power field, consistent with an active UTI. Antibiotic treatment days averaged 196 ± 100 days for the Decentralized animals and 221 ± 45 days for ObNT Reinn animals.

### KCl-, EFS-, and ATP-Induced Contractions

**Figure 2.**
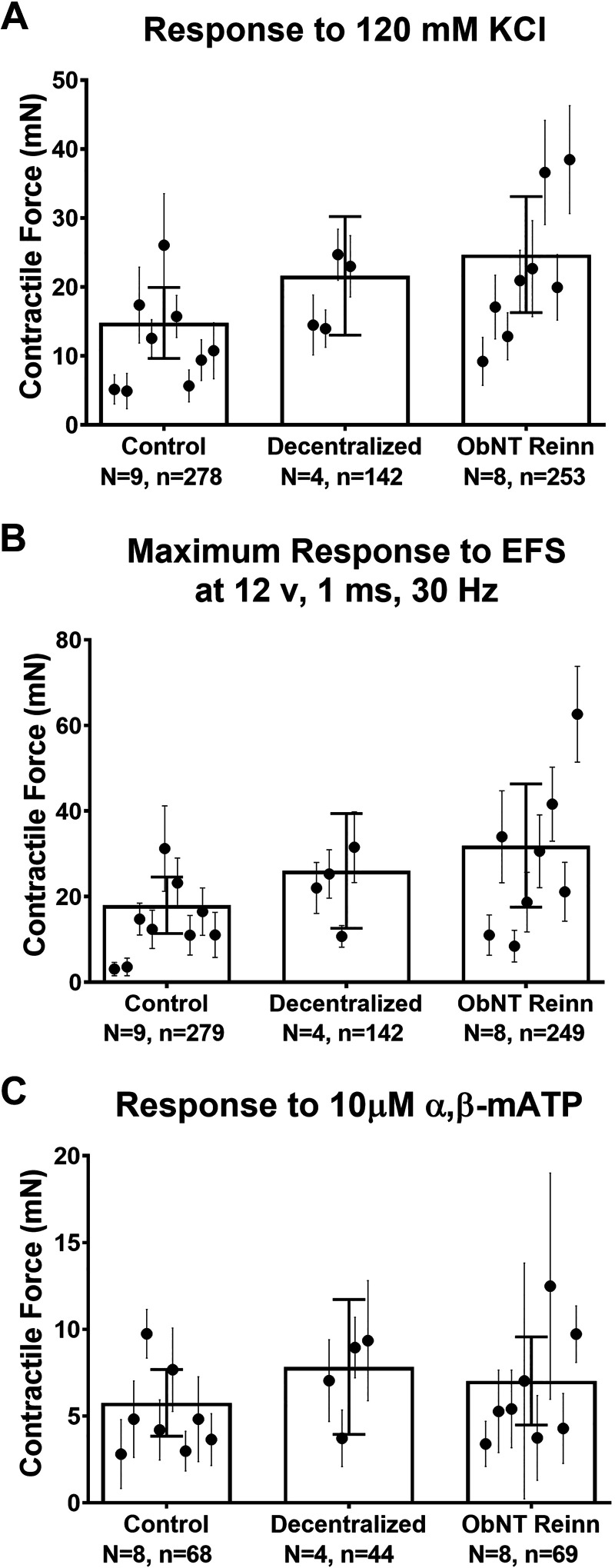
Smooth muscle strip contractility. Mucosa denuded bladder strips isolated from control (sham-operated and unoperated), Decentralized and ObNT Reinn canines. Contractile responses to high concentration of extracellular potassium (120 mM KCl) (*A*), electric field stimulation (EFS) of 12 V, 1 ms pulse duration 30 Hz frequency (*B*), and 10 μM α,β-mATP (*C*). Responses are expressed in milli Newtons (mN). KCl, potassium chloride; *N*, number of dogs per group; *n,* total number of muscle strips per group. Closed circles represent means ± 95% CIs for the strips from individual animals and bars represent means ± 95% CIs of the means of the strips from the individual animals. CI, confidence interval.

After equilibration of the muscle strips to the bathing solution, we observed very few spontaneous contractions >1–4 mN and there was no difference in spontaneous activity between treatment groups. Bladder smooth muscle strips free of mucosa contracted with exposure to Tyrode’s solution containing 120 mM KCl. The induced maximal contractile forces were not statistically significantly different across groups (Fig. 2*A*). Upon stimulation with an electric field of 12 V, 1 ms pulse duration, and increasing frequencies (2, 5, 12, 20, and 30 Hz), the maximal EFS-evoked contractions did not differ across groups (*P* = 0.10, Fig. 2*B*). No differences between groups were observed in the contractile response to activation of purinergic receptors with 10 μM α,β-mATP (*P* = 0.42, Fig. 2*C*). Responses were determined to a single 30-μM concentration of the muscarinic receptor agonist bethanechol in bladder strips of three Decentralized (*N* = 3, *n* = 63) and five ObNT Reinn (*N* = 5, *n* = 113) animals and those responses were similar in both groups (data not shown). Responses measured as means ± 95% CI in Decentralized and ObNT Reinn groups were 37.5 mN (95% CI: 29.4–45.3) and 41.9 mN (95% CI: 26.5–55.2), respectively. Response to 30 µM bethanechol was also tested in normal male dogs (*N* = 3, *n* = 9) measured as means ± 95% CI was 20.5 mN (95% CI: 0.0–55.6).

Collectively, these data show that after long-term decentralization and reinnervation, the contractile properties of bladder smooth muscle strips are preserved in that muscle cells have excitable membranes and functional contractile elements indicated by their responses to KCl. Also, motor nerve endings are still sensitive to the EFS activation by releasing the neurohumoral transmitters ACh and ATP.

### Altered Electrical Excitability of Bladder Nerve Endings in Decentralized and ObNT Reinn Bladders

**Figure 3.**
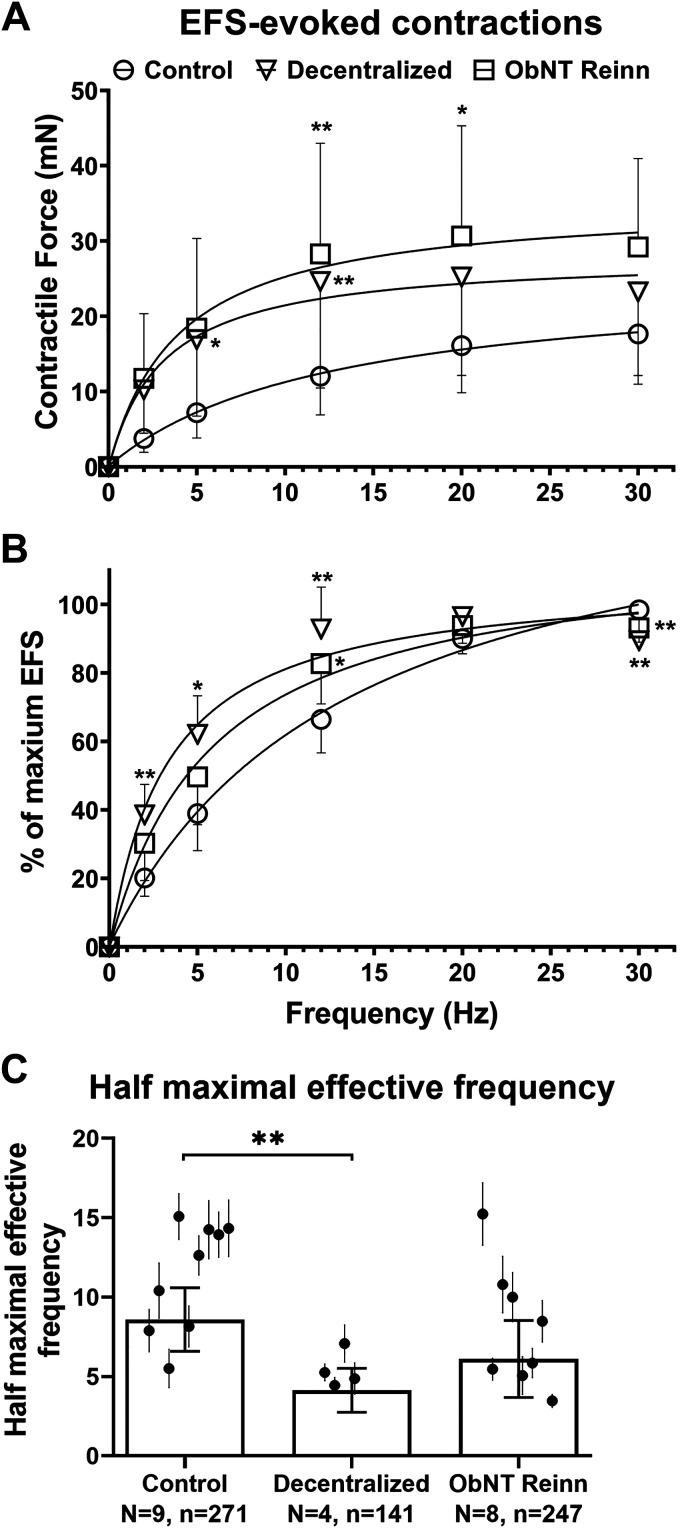
Frequency response curves were generated for each strip at varying frequencies (2, 5, 12, 20, and 30 Hz), 12 V and 1 ms pulse duration. Responses in the presence of vehicle (H_2_O) are presented in milli Newtons (mN) (*A*) and as a percent of maximum EFS (*B*). *C*: calculated half maximal effective frequency. *N*, number of dogs per group; *n*, total number of muscle strips per group. Data are presented as means ± 95% CI. *P < 0.05 and **P < 0.01 compared with control group. CI, confidence interval.

Frequency response curves of the EFS-evoked contractions were different between groups for both force of contraction and percent of maximum EFS (Fig. 3, *A* and *B*, respectively). A repeated-measures mixed-effects model analysis for force changes showed an effect for frequency, surgical group, and frequency × surgical group interaction (*P* ≤ 0.0001, *P* = 0.03, and *P* ≤ 0.0001, respectively, Fig. 3*A*). A repeated-measures mixed-effects model analysis for percent of maximum EFS showed effects for frequency, surgical groups, and frequency × surgical group interaction (*P* ≤ 0.0001, *P* ≤ 0.0001, and *P* ≤ 0.0001, respectively, Fig. 3*B*). Strips from Decentralized and ObNT Reinn bladders showed a shift to the left in the frequency-response curves with increased sensitivity to EFS at lower frequencies compared with strips from normal control bladders (Fig. 3, *A* and *B*). The calculated half-maximally effective frequency (EF_50_) of the nerve-evoked contractions were different across groups (*P* = 0.0006, Fig. 3*C*). Post hoc analyses showed that the nerve-induced contractions exhibited a decrease in EF_50_ values in Decentralized strips compared with normal control strips (*P* = 0.004, Fig. 3*C*). EF_50_s of strips from ObNT Reinn bladders were not statistically significantly different than either the Decentralized or normal control strips (Fig. 3*C*). This shows increased excitability to EFS in Decentralized bladder strips and bladder reinnervation lowers that excitability toward the control level.

### Effect of Purinergic and Cholinergic Blockade on EFS-Induced Frequency-Response Curves and Alterations in the Purinergic Component of EFS-Induced Contractility

**Figure 4.**
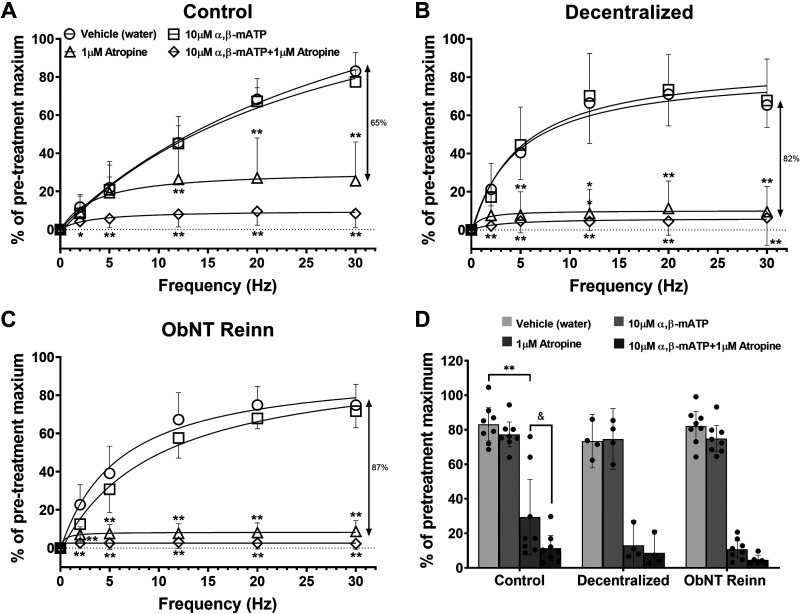
Effect of blockade of purinergic and cholinergic components with α,β-mATP and atropine, respectively, as well as both α,β-mATP and atropine on the maximal nerve-evoked contractions. Frequency response curves were generated for each strip at varying frequencies (2, 5, 12, 20, and 30 Hz), 12 V, and 1 ms pulse duration. Responses are presented as percent of maximal electric field stimulation (EFS)-induced contractions prior to treatments with either vehicle (H_2_O), 10 μM α,β-mATP, 1 μM atropine, or 10 μM α,β-mATP + 1 μM atropine. The double-headed arrows indicate the atropine-sensitive contractions in each group. Only the maximal responses expressed as the precent of pretreatment maximum are plotted in *D* to show differences between groups. Number of animals per group: control (sham-operated and unoperated) = 8 (*A* and *D*), Decentralized = 4 (*B* and *D*), and ObNT Reinn = 8 (*C* and *D*). Numbers of strips per treatment group for control, Decentralized, and ObNT Reinn, respectively, are vehicle (46, 30, and 48), α,β-mATP (40, 27, and 41), atropine (38, 29, and 47), and α,β-mATP + atropine (50, 27, and 29). Data are presented as means ± 95% CI. *P < 0.05 and **P < 0.01 compared with vehicle. &*P* < 0.05 compared with atropine treatment in control group. CI, confidence interval.

Desensitization of purinergic receptors with 10 μM of the purinergic receptor agonist α,β-mATP did not block the maximal nerve-evoked contractions in bladder strips from normal control, Decentralized, or ObNT Reinn animals compared with vehicle treatment, regardless of the frequency tested [Fig. 4, *A*–*C* (□)]. Figure 4*D* shows that less than 9% of the maximal evoked contractions were blocked by α,β-mATP in the three surgical groups. In contrast, 1 μM of the nonsubtype selective muscarinic receptor antagonist atropine effectively inhibited these contractions in the three groups compared with vehicle treatment and the inhibition was greater in the Decentralized and ObNT Reinn groups compared with normal control group, (Δ in Fig. 4, *A*–*C*). In normal control bladder strips, ∼65% of the maximal nerve-evoked contractions were atropine-sensitive; the mean maximum response as percentage of pretreatment for postatropine versus postvehicle was 29.44% (95% CI: 7.63%–51.25%) versus 83.16% (95% CI: 73.56%–92.76%; Fig. 4*A*]. However, the sensitivity of contractions to atropine with respect to vehicle increased to ∼82% in the Decentralized [13.06% (95% CI: −1.48% to 27.60%] vs. 73.42% (95% CI: 57.90%–88.93%); Fig. 4*B*) and 87% in the ObNT Reinn bladder strips [10.77% (95% CI: 5.83%–15.72%) vs. 82.02% (95% CI: 73.35%–90.70%); Fig. 4*C*]. A repeated-measures mixed-effects model analysis showed an effect for drug treatment (P ≤ 0.0001, Fig. 4*D*). Similarly, blockade with α,β-mATP plus atropine caused a similar inhibition of the EFS-evoked contractions in all groups (86%, 88%, and 94%, respectively, ◊ symbols in Fig. 4, *A*–*C*). However, the inhibition by atropine was further inhibited by the addition of α,β-mATP in normal control bladder strips compared with atropine alone, but not in Decentralized or ObNT Reinn bladder strips (Fig. 4*D*). A repeated measures mixed-effects model analyses showed an effect for drug treatment (*P* ≤ 0.0001, Fig. 4*D*). Post hoc analyses showed an increase in the inhibition of the maximal evoked contractions by α,β-mATP plus atropine in the normal control (*P* = 0.015, Fig. 4*D*).

**Figure 5.**
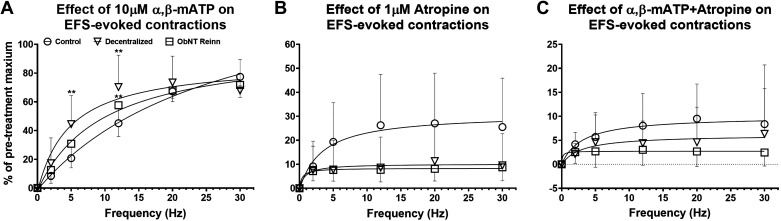
Blockade of purinergic and cholinergic components using 10 μM α,β-mATP and 1 μM atropine, respectively. Frequency response curves were generated for each strip at varying frequencies (2, 5, 12, 20, and 30 Hz), 12 V, and 1 ms pulse duration. Responses are presented as percent of maximal electric field stimulation (EFS)-induced contractions before treatments with either 10 μM α,β-mATP (*A*), 1 μM atropine (*B*), or 10 μM α,β-mATP + 1 μM atropine (*C*). Number of animals per group: control (sham-operated and unoperated) = 8, Decentralized = 4, and ObNT Reinn = 8. Numbers of strips per treatment per group, respectively, are α,β m-ATP (40, 27, and 41), atropine (38, 29, and 47), and α,β-mATP + atropine (50, 27, and 29). Data are presented as means ± 95% CI. **P < 0.01 compared with control. CI, confidence interval.

The previous data presented in Fig. 4, *A*–*C* is reorganized in Fig. 5, *A*–*C* to show changes in responses to drug treatments. When purinergic receptors are desensitized with 10 μM α,β-mATP, the cholinergic components mediate ∼75%–77% of the maximal nerve-evoked contractions in the three surgical groups; normal control, Decentralized, and ObNT Reinn. A repeated-measures mixed-effects model analysis showed an effect for frequency and frequency × surgical group interaction (*P* ≤ 0.0001 and *P* ≤ 0.0001, respectively, Fig. 5*A*). In strips from Decentralized and ObNT Reinn bladders, the cholinergic neurotransmission was more prominent at frequencies of 5 and 12 Hz compared with control. On the other hand, upon treatment with 1 μM atropine, the purinergic components mediate ∼27% of the maximal nerve-evoked contractions in normal control bladder strips, but only 11% in Decentralized and 9% in ObNT Reinn bladder strips (Fig. 5*B*). A repeated-measures mixed-effects model analysis showed an effect for surgical group (*P* = 0.002 and *P* = 0.002, Fig. 5*B*). After the treatment with α,β-mATP and atropine combined, only 10%, 6%, and 3% of the maximal nerve-evoked contractions remained in the strips from control, Decentralized, and ObNT Reinn bladders, respectively (Fig. 5*C*), indicating that, in all groups, 90%–97% of the responses are mediated by a combination of cholinergic and purinergic components. A repeated-measures mixed-effects model analysis showed an effect for surgical group (*P* = 0.001, Fig. 5*C*). Therefore, bladder decentralization and reinnervation have no apparent effect on the cholinergic component of the EFS (i.e., ACh is still released from nerve endings). Although, the sensitivity of the evoked contractions to atropine increased in Decentralized and ObNT Reinn bladders, ACh and ATP are the main neurotransmitters that mediate the effect of EFS-evoked contractions.

### Changes in EFS-Induced Contractility after Treatment with TTX

**Figure 6.**
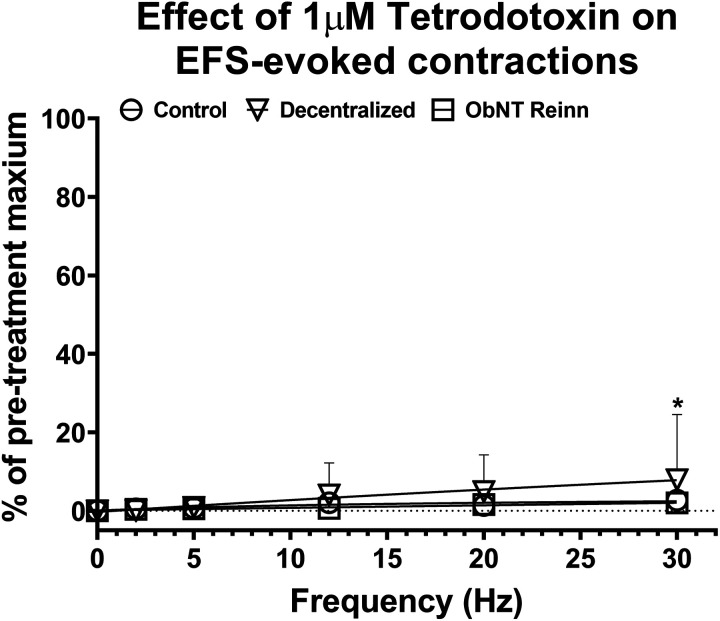
Blockade of nerve-evoked contractions using 1 μM tetrodotoxin. Frequency response curves were generated for each strip at varying frequencies (2, 5, 12, 20, and 30 Hz), 12 V, and 1 ms pulse duration. Responses are presented as percent of maximal electric field stimulation (EFS)-induced contractions before treatment with 1 μM tetrodotoxin. Number of animals per group: control (sham-operated and unoperated) = 9, Decentralized = 4, and ObNT Reinn = 8. Number of strips per group, respectively, are 47, 22, and 42. Data are presented as means ± 95% CI. *P < 0.05 compared with control. CI, confidence interval.

In the three experimental groups, the sodium channel blocker tetrodotoxin (TTX) nearly eliminated EFS-induced contractions (Fig. 6). A repeated-measures mixed-effects model analysis showed an effect for frequency and frequency × surgical group interaction (*P* < 0.0001 and *P* = 0.0004, respectively, Fig. 6). In control strips, 1 μM TTX effectively inhibited ∼97%–99% of field stimulations at all frequencies used (Fig. 6). Although TTX greatly reduced electrically evoked response at all frequencies tested in Decentralized bladder (Fig. 6), the mean maximum response at 30 Hz was reduced by only 92%, with respect to the control maximum. There was no difference in the elimination of maximal contractions between ObNT Reinn bladder strips (Fig. 6) and control strips. Thus, EFS-induced contractions in strips from Decentralized and ObNT Reinn bladders through releasing ACh and ATP from intramural nerve endings and those contractions are mostly TTX sensitive. Yet, strips from Decentralized bladder develop resistance to the toxin concomitant with increased signs of inflammation.

## DISCUSSION

The goal of this study was to further investigate the effects of both decentralization via transection of all spinal roots, as well as hypogastric nerves, contributing to normal bladder innervation and surgical reinnervation on bladder smooth muscle function using ex vivo contractility studies. Changes in the contribution of muscarinic or purinergic receptor-mediated activity in the detrusor smooth muscle free of mucosa was assessed.

The pilot study of this series (17) shows that transection of all sacral roots plus hypogastric nerves was not enough to eliminate squat-and-void postures and that the additional L7 dorsal root transection is necessary to eliminate those behaviors in ∼57% of the decentralized dogs. The new neuronal pathways created by bladder reinnervation via obturator-to-pelvic nerve and sciatic-to-pudendal nerve transfer surgeries recovered both sensory and motor functions after long-term decentralization. Part 2 of this series (23) shows that long-term bladder decentralization or delayed reinnervation results in loss of urothelial integrity, increased signs of inflammation in the submucosal and detrusor layers and increased bladder wall thickness, specifically the muscle layer due to increased collagen deposition without a change in the percent muscle area. Reduced intramural ganglia numbers and cells are observed in bladder wall of decentralized and reinnervated dogs. Although axon density declines in decentralized bladders, it increases to control level in reinnervated bladders. Correlation studies indicate that although enhanced inflammation and fibrosis impair bladder function, increased axonal density after reinnervation may help the bladder to retain its function.

Continued responsiveness to KCl after decentralization (Fig. 2*A*) suggests preservation of smooth muscle contractility, as previously reported (21). It is of interest that the extensive removal of spinal roots did not deteriorate bladder smooth muscle responsiveness to stimulatory agents after one year because smooth muscle function is a necessary component of bladder physiology as a whole and would need to be present for successful surgical reinnervation. It is possible that the nonstatistically significantly enhanced responses to KCl in strips from ObNT Reinn bladders might be indicative of developing super sensitivity, although no studies to date have explored this possibility. A previous study reported that detrusor strips from patients with overactive bladders were supersensitive to KCl as a result of parasympathetic decentralization (24).

The response to electrical field stimulation in strips from spinal root transected bladders (Fig. 2*B*) indicates that neurotransmitters mediating contractions are released from intramural nerve endings that innervate the detrusor muscle. No change in the ex vivo response to nerve stimulation indicates that the postganglionic innervation of the bladder wall is still intact (21). In ObNT Reinn bladders, perhaps the nerve transfer procedure enhances the neurotransmitter release from nerves that are running out of the intramural ganglia in the bladder wall (20). According to an earlier study, alterations of sensory stimuli and chronic surgical denervation or decentralization can induce postjunctional super sensitivity. In addition, a decrease in cholinesterase and an increase in electrical excitability might contribute to this phenomenon (25). In contrast, a different investigation suggested that the reduction in the activity of the excitatory nerves, due to a partial denervation of the detrusor, might be responsible for the alterations in the muscle properties including an increase in the excitability and the emergence of myogenic contractions (26). Further investigation might help in understanding the underlying mechanisms that contribute to enhanced muscle sensitivity and excitability. Similarities in the transient contractions of bladder strips from control, Decentralized, and ObNT Reinn animals in response to the purinergic agonist (Fig. 2*C*) demonstrate the presence of functional purinergic receptors on bladder smooth muscle cells, regardless of surgical treatment condition (27).

The data in Fig. 3 demonstrate that smooth muscle strips from Decentralized bladders were stimulated to contract more easily at lower frequencies, reflected by a reduction in the EF_50_ (Fig. 3*C*). We speculate that elimination of the innervation to the pelvic plexus and bladder wall increases smooth muscle nerve endings’ excitability to EFS. A study of normal human bladder reported that EFS-induced atropine-resistant contractions were markedly enhanced at low-frequency stimulations (28). A different study reported that bladder muscle samples from patients with outflow obstruction, neurological lesions or idiopathic uninhibited bladder conditions and from patients with neurogenic bladders tended to show a left shift of the electrically stimulated force-frequency response (2, 5, 29). According to other groups, the reduction in the maximal active force and the shift in frequency of stimulation to produce maximum contraction could be due to changes that occur following decentralization such as changes in bladder wall morphology, nerve degeneration, changes in transmission output, or increased bladder sensitivity to contractile agents (30, 31). The enhanced contractile response at low frequencies in the Decentralized and ObNT Reinn bladder strips could be due to morphological changes that might have occurred as a result of extensive long-term decentralization. Part 2 of this series of studies shows that the bladder wall thickness and the detrusor muscle layer are thicker in Decentralized and ObNT Reinn animals than in control (23). Although, decentralization and reinnervation results in increased collagen in the bladder wall, the percent area with muscle in the detrusor layer is not different across groups. Because the percent muscle of the detrusor is maintained after long-term decentralization and reinnervation, this supports preservation of the bladder contractile properties, as we previously reported (21). Further investigation of changes in the bladder wall following extensive decentralization and delayed reinnervation are discussed in more detail in part 2 of this study (23).

We previously found that denervation or decentralization of the rat bladder leads to considerable bladder hypertrophy (32, 33). The innervation of the rat bladder is somewhat unique in that the bladder wall is devoid of neuronal ganglia. Neuronal cell bodies innervating the rat bladder exist outside the bladder wall, primarily in the major pelvic ganglion (MPG). This is not so for the canine bladder and other species including human in which neuronal cell bodies and ganglia are prominent in the bladder wall itself. Within 24 h after electrocautery of the rat major pelvic ganglion, the in vitro response of bladder muscle strips to electric field stimulation is markedly reduced but not if the rat bladder is decentralized by severing all nerve fibers entering the MPG from the spinal cord. The response to the cholinergic agonist carbachol was found to be unchanged in denervated or decentralized rat bladders compared to sham-operated animals (32, 33). Thus, one could conclude that in decentralized or denervated bladders, as bladder strips of similar size contract comparably to muscarinic receptor stimulation but overall bladder mass increases, the potential exists for increased pressure of the whole bladder with sufficient muscarinic and perhaps purinergic receptor stimulation.

Although cholinergic receptor-mediated activity makes up the majority of EFS-evoked smooth muscle contraction across groups, purinergic receptor-mediated contractions are reduced in Decentralized and ObNT Reinn bladders (Fig. 4). It has been reported that the contribution of cholinergic and purinergic components to bladder contraction varies among species and pathological conditions (6, 34, 35). An increased involvement of the cholinergic component, and to a lesser extent purinergic component, in the EFS-evoked contractions was reported in rat bladder after neonatal sensory denervation with capsaicin (36). The enhanced atropine sensitivity of EFS-induced contractions in the Decentralized and ObNT Reinn bladder strips agrees with a study in bladder strips from long-term spinal cord-transected rats (8). However, another study indicated that although the EFS-induced contractions in strips of human bladder samples obtained from patients with bladder cancer or stress incontinence but no urge symptoms were almost entirely atropine-sensitive, a cholinergic contribution accounted only partially for the EFS-induced contractions in samples from patients with functionally disturbed bladders that showed large volumes of residual urine and uninhibited bladder contractions on filling urodynamics (7, 29). In mammalian bladders other than human, studies reported that alterations in cholinergic transmission could be due to the release of additional minor activators such as prostaglandins from the tissue, which either modulate the postsynaptic response to acetylcholine or change the adrenergic transmission (37, 38). Although, in this current study, we did not test this prospect, further investigation might be necessary to rule out any similar possibility.

Although it is reported that the purinergic response decreases with pathology in a rat model of spinal cord injury-induced bladder dysfunction (8, 39), it increases following pathology in human bladders (3, 29). Conflicting results relating to atropine-resistant contractions and purinergic transmission have been reported in human urinary bladder strips. Some reports indicate that patients with interstitial cystitis, detrusor instability, or neurogenic bladders exhibit an increase in the magnitude of atropine-resistant bladder contractions compared with healthy patients (3–5, 40). However, an absence of atropine-resistant contractions in normal human bladders has also been reported (41). Although atropine-resistant contractions were shown to be mediated by ATP-acting on purinergic receptors in normal bladder smooth muscle (35), no purinergic components were shown to be involved in nerve-evoked contractions in other studies of normal human bladders (5, 41). Studies of unstable bladder syndromes reveal abnormalities in purinergic transmission as well as purinergic receptor subtype expression (3, 4). The percentage of nerve-mediated contraction remaining after desensitizing purinergic receptors with α,β-mATP followed by atropine application (Fig. 4) is only slightly above the residual response observed after blocking sodium channels with tetrodotoxin (Fig. 6), suggesting that neuronally released ACh and ATP accounts for nearly all of the EFS induced contractile responses. The small (<10%) residual contraction that remained after α,β-mATP plus atropine treatment was not different between groups.

The EFS-evoked contractions in the three groups appear to be nerve-mediated because they disappear with the sodium channel blocker, tetrodotoxin (Fig. 6). The EFS-induced contractions that remained after TTX treatment in the Decentralized bladder strips at higher frequencies (12% of the maximum response) could perhaps result from direct stimulation of the muscle (24) or TTX resistant release of a noncholinergic, nonpurinergic agent such as prostaglandins or other unknown contractile agents (42, 43). Worthy of note is that the TTX-resistant contractions were mainly noticed in a dog bladder that exhibited increased signs of inflammation. It has been reported that acute inflammation, including UTI, alters bladder function by the release of different inflammatory mediators responsible for bladder wall irritation and other pathological changes (44). Supportive data will be discussed in more detail in part 2 of this study (23). Recurrent inflammation and durable release of those noxious stimuli may influence nerve excitability by changing the expression of sodium channel isoforms on those nerves (45). Therefore, further examination is required to understand the mechanism underlying the relationship between TTX-resistant contractions and increased inflammation in Decentralized bladders.

The reduction in the purinergic component of nerve-evoked contractions, is not supported by the fact that α,β-mATP desensitization did not suppress EFS contractions but it is supported by the increased inhibition by atropine and the lack of further inhibition by the combination of atropine and α,β-mATP in Decentralized and ObNT Reinn bladder strips. This reduction may be due to either reduced ATP release from nerve terminals or increased ecto-ATPase activity at the synapse. Ecto-ATPase disables the initial crucial steps in the nerve-evoked cascade that leads to elevated intracellular Ca^2+^ levels and contraction of smooth muscle (29, 46). As ATP acts directly upon purinergic receptors inducing Ca^2+^ influx, it is expected that the impaired ATP release would attenuate smooth muscle membrane depolarization in canines with eliminated innervation to the pelvic plexus and bladders. As strips from Decentralized bladders exhibited responses to EFS similar to controls and even greater responses at lower frequencies, the possibility exists that there may be additional excitatory neurotransmitters besides acetylcholine and ATP that may take part in the initiation of the action potential at the nerve terminals. Similar findings were previously reported (47, 48). Changes in bladder innervation via decentralization may alter the amount of ATP and acetylcholine released from bladder smooth muscle nerves, which could be tested by measuring the amounts of neurotransmitters released during EFS-induced contractions. Thus, further investigation is warranted.

This study did not include concentration-response curves for a muscarinic agonist such as bethanechol, however we found no differences in the robust contractile responses to 30 μM bethanechol between groups. Thus, although we cannot discern possible differences in bethanechol potency between surgical groups from the responses to this single, essentially maximally effective concentration, if changes in bladder muscle muscarinic receptor density were induced by the decentralization and reinnervation surgeries, these changes did not affect the maximal response to muscarinic receptor stimulation.

### Perspectives and Significance

In summary, this study shows that alterations in bladder innervation in canines, by either extensive spinal root transection alone or nerve rerouting after long-term decentralization, increases sensitivity to nerve evoked muscle contractile responses to electric field stimulation. Although contractile responses increase with increasing frequency, responses are shifted to a higher magnitude at low frequencies in Decentralized bladders collected 11–21 mo postdecentralization and in ObNT Reinn bladders collected 8–12 mo postreinnervation surgeries. These ex vivo studies showed that bladder smooth muscle preserves its contractility even after long-term decentralization. Despite the reduction in the neuronal inputs due to extensive decentralization, intramural nerve endings are able to release their neurotransmitters in response to electric field stimulation, and they become more sensitive to the stimulation. One of the major findings in this study is a dominant contribution of cholinergic components to neuronal contractions in Decentralized and ObNT Reinn canine bladders; yet purinergic components mediated only a small proportion of nerve-evoked contractions.

## GRANTS

Research reported in this publication was supported by the National Institute of Neurological Disorders and Stroke of the National Institutes of Health under Award Number R01NS070267 (to M.R.R. and M.F.B.). The high definition video cystoscopy system used for the retrograde dye injections was supplied from a Customer Initiated Equipment Grant from Karl Storz Endoscopy-America, Inc., El Segundo, CA.

## DISCLAIMERS

The content is solely the responsibility of the authors and does not necessarily represent the official views of the National Institutes of Health.

## DISCLOSURES

Michael R. Ruggieri, Sr. received the Video Cystoscopy Equipment Grant from KarlStorz America. None of the other authors has any conflicts of interest, financial or otherwise, to disclose.

## AUTHOR CONTRIBUTIONS

N.F., A.S.B., D.S.P., J.M.B., M.M., M.F.B., and M.R.R. conceived and designed research; N.F., D.G., A.S.B., D.S.P., J.M.B., M.M., I.J.W., M.A.P., E.T., C.L.T., L.J.H., M.F.B., and M.R.R. performed experiments; N.F., D.G., A.S.B., D.S.P., J.M.B., D.Y., M.F.B., and M.R.R. analyzed data; N.F., D.G., A.S.B., D.S.P., J.M.B., M.F.B., and M.R.R. interpreted results of experiments; N.F., D.G., and M.F.B. prepared figures; N.F. and D.G. drafted manuscript; N.F., D.G., D.S.P., J.M.B., M.F.B., and M.R.R. edited and revised manuscript; N.F., D.G., A.S.B., D.S.P., J.M.B., M.M., I.J.W., M.A.P., E.T., C.L.T., D.Y., L.J.H., M.F.B., and M.R.R. approved final version of manuscript.
